# Integrative AI-metabolomics: a new frontier in diagnosing pulmonary tumor thrombotic microangiopathy

**DOI:** 10.1097/MS9.0000000000003707

**Published:** 2025-08-13

**Authors:** Fahad Amin, Hira Khalid, Maliha Khalid, Muhammad Talha, Aminath Waafira

**Affiliations:** a Departmant of medicine, King Edward Medical University, Lahore, Pakistan; b Department of Molecular Biology and Genetic, Liaquat University of Medical and Health Sciences, Jamshoro, Pakistan; c Departmant of medicine, Jinnah Sindh Medical University, Karachi, Pakistan; d School of Medicine, The Maldives National University, Malé, Maldives

**Keywords:** artificial intelligence, machine learning, metabolomics, pulmonary tumor thrombotic microangiopathy

## Abstract

Pulmonary tumor thrombotic microangiopathy (PTTM) is a rare, rapidly progressive malignancy-related pulmonary vascular condition that remains difficult to diagnose before death due to its nonspecific clinical and imaging features. Recent advances in metabolomics, particularly when powered by artificial intelligence (AI), offer promising opportunities for early detection by identifying distinct metabolic signatures. While AI-driven metabolomics has shown success in various cancers, its application to PTTM is challenged by the rarity of the disease, limited datasets, and privacy concerns. Innovations such as multicenter collaborations and blockchain-based data sharing may help overcome these barriers. Integrating AI and metabolomics has the potential to revolutionize not only the diagnosis of PTTM but also other rare diseases with elusive early biomarkers.

*To the Editor,*

Pulmonary tumor thrombotic microangiopathy (PTTM) is a malignancy-associated condition characterized by embolization of neoplastic cells to the pulmonary vasculature. This results in fibro-cellular intimal proliferation, activation of the coagulation cascade, and high-grade pulmonary hypertension. Patients develop progressive dyspnea and hypoxia and may die within a matter of days due to right-sided heart failure. Ante-mortem diagnosis is complicated due to a lack of definitive symptoms and imaging findings. While a biopsy can confirm the diagnosis, it remains high-risk in unstable patients. A recent review showed that incorporating pulmonary artery catheter cytology or FDG-PET may allow timely intervention and better outcomes^[1]^.

Metabolomics studies metabolites within cells, tissues, or organisms and provides a live snapshot of biochemical activity, indicating cellular function and physiological state using devices such as mass spectrometry, nuclear magnetic resonance, chromatography, and chemometrics. Machine learning (ML) has advanced the field of metabolomics by identifying micro-metabolic changes and complex patterns. ML is important in identifying novel biomarkers and making future models for disease diagnosis and treatment. A key application is feature selection, which reduces large datasets to relevant variables. ML-powered metabolomics has highlighted biomarkers in cancers such as colorectal, ovarian, kidney, and pancreatic cancer. Integrating metabolomics with other -omics data has increased cancer classification and enabled individual medical strategies^[2]^.

As PTTM is a rare but fatal condition mainly diagnosed after death during post-mortem, with a poor survival rate^[3]^. There is an urgent need for better early diagnostic approaches. Metabolomics can be a promising approach that offers early diagnosis, as it can identify specific metabolic signatures linked with the progression of PTTM. It analyzes normal metabolite levels in the body and detects alterations in biochemical reactions in the body^[4]^. Potential biomarkers can enable early diagnosis. Integrating artificial intelligence (AI) into metabolomics enhances data collection and analysis, facilitating the elucidation of complex metabolic pathways and enabling more effective multiomics integration^[5]^.

Despite these advancements, applying AI-driven metabolomics to PTTM faces several challenges. As mentioned, PTTM is a rare condition, and its true prevalence is unknown^[3]^. This limits the availability of samples, creating a hurdle for detailed metabolomics studies and the training of robust AI models^[4]^. Additionally, variations in metabolic measurements across different populations complicate data integration^[5]^. The reliance on large datasets raises significant concerns about data privacy, security, and authenticity. This also increases the risk of misuse if the data is not well protected. Although blockchain technology has emerged as a potential solution to these challenges, several obstacles remain to its implementation^[6]^. Our work is in line with the TITAN Guidelines on the need for transparency in AI use in healthcare^[7]^.

In conclusion, AI-driven Metabolomics profiling holds the promising potential for the early detection of PTTM, although several challenges, such as small sample size and some technical barriers, exist. Innovative strategies are required, such as collaborations between multiple centers and data sharing by blockchain technology, to overcome these hurdles. Future research is needed to assess potential metabolomics biomarkers and develop more accurate AI models that can differentiate PTTM from other causes of pulmonary hypertension. These strategies will not only help in the early diagnosis of PTTM but can also improve the diagnosis of other rare diseases.

## Data Availability

No data were generated for this manuscript.
